# Initial intravitreal injection of high-dose ganciclovir for cytomegalovirus retinitis in HIV-negative patients

**DOI:** 10.1186/s12886-018-0983-z

**Published:** 2018-12-10

**Authors:** Zhuyun Qian, Haili Li, Yong Tao, Wensheng Li

**Affiliations:** 1 Shanghai Aier Eye Hospital, Shanghai, China, No. 1286, Hongqiao Road, Changning District, Shanghai, 200050 China; 2 Department of Ophthalmology, the First Hospital of Peking University, Beijing, China; 3 Department of Ophthalmology, Beijing Chaoyang Hospital, Capital Medical University, No. 8, South Road of Worker’s Stadium, Chaoyang District, Beijing, 100020 China; 4 Aier School of Ophthalmology, Central South University, Changsha, Hunan Province China

**Keywords:** Cytomegalovirus retinitis, High-dose, Ganciclovir, Intravitreal injection, Aqueous humour

## Abstract

**Background:**

The purpose of this study is to examine the clinical outcomes achieved by using initial high-dose intravitreal ganciclovir injections to treat cytomegalovirus retinitis in patients without human immunodeficiency virus (HIV) infection.

**Methods:**

Twenty-four eyes (24 patients) with cytomegalovirus retinitis received multiple intravitreal injections of ganciclovir in weekly intervals. A higher dose (6 mg) of ganciclovir was applied at the first intravitreal injection, and a lower dose was used for maintenance. Anterior aqueous humour was obtained before each injection. The best-corrected visual acuity and cytomegalovirus loads in the anterior aqueous humour were measured.

**Results:**

The mean cytomegalovirus DNA load in aqueous humour decreased significantly from (2.59 ± 2.28) × 10^5^ copies/mL at baseline to (1 ± 1.76) × 10^4^ copies/mL one month later. The aqueous cytomegalovirus DNA load was negative in 17 eyes (70.8%) one month later. No obvious improvement of best-corrected visual acuity was found during the treatment. A positive correlation was proven between initial cytomegalovirus DNA titers in aqueous humour and the total number of intravitreal injections of ganciclovir, as well as between the baseline and final best-corrected visual acuities. No severe complications developed.

**Conclusions:**

An initial high dose of ganciclovir (6 mg) and continuous intravitreal injections of ganciclovir could significantly decrease the cytomegalovirus load in HIV-negative patients with cytomegalovirus retinitis.

**Trial registration:**

http://clinicaltrials.gov, NCT03598452, retrospectively registered on 24 July 2018.

Cytomegalovirus (CMV) infection is common in the general population [1]. Nearly 60% of individuals over the age of 60 and more than 80% of those over the age of 80 exhibit seropositivity [2]. CMV infection remains asymptomatic in people with healthy immune systems, but in immunocompromised individuals (such as those with acquired immunodeficiency syndrome [AIDS], pharmacologic immunosuppression after organ grafting or hematologic malignancy, etc), it is a major cause of morbidity and mortality.

Active intraocular CMV infection leads to severe retinal destruction and poor prognosis if left untreated. CMV retinitis (CMVR) is classically associated with AIDS, but reports of CMVR in HIV-negative populations continue to increase due to the development of modern immunosuppressive therapies and improved survival rates [3, 4].

Diagnosis of CMVR is based on clinical symptoms and confirmed by the detection of CMV in an aqueous or vitreous samples by polymerase chain reaction (PCR) [5]. The first-line treatment of CMVR includes the systematic and topical use of ganciclovir. Foscarnet is considered in cases in which ganciclovir is intolerable or ineffective. Intravitreal antiviral drug delivery was used as the first-line treatment in several studies in which systematic injection had been ruled out [6–8]. The reported dose of intravitreal injections of ganciclovir (IVG) varied from 200 μg/0.1 mL to 5 mg/0.1 mL in patients with AIDS [9]. Our previous work showed the safety and the efficacy of 1 mg IVG in HIV-negative patients suffering from CMVR; the percentage of eyes in which CMV could not be detected rose with each consecutive injection. We proposed that a higher dose of ganciclovir in the first injection followed by lower maintenance doses would indicate a better result [10]. Therefore, this study was performed to evaluate the safety and the potential therapeutic effect of the initial high-dose IVG for CMVR in HIV-negative patients.

## Methods

In the last four years, 26 HIV-negative patients were diagnosed with CMVR through ophthalmological examination and positive CMV DNA in aqueous humour by real-time PCR in department of ophthalmology, Beijing Chaoyang Hospital. All patients were identified using electronic medical records. Two of those patients had diabetic retinopathy and glaucoma and were excluded from this study. So, this clinical interventional case-series included 24 continuous patients with CMVR, which was consecutively treated by intravitreal injections of ganciclovir.

Among all studied patients, nine had bilateral ocular disease. To avoid the non-independence of eyes from the same person, data were collected in a randomly selected unilateral eye from each patient. The off-label use of the drug and its potential risks and benefits were discussed in detail with all patients, who then signed formal consents. The study was conducted in accordance with the Declaration of Helsinki. Institutional review board approval was obtained.

The initial dose was 6 mg/0.1 mL of ganciclovir; it was reduced to 4.5 mg/0.1 mL in the second injection, and 3 mg/0.1 mL of IVG was used as a maintenance dose. The injections were stopped at the discretion of the treating doctor based on CMV titers and an assessment of clinical efficacy and/or futility. The injection method was the same as we described in our previous reports [10]. Before each injection, 50 μl of anterior aqueous humour was obtained through paracentesis at seven o’clock, and real-time PCR was applied immediately to detect the load of aqueous CMV DNA level. In cases in which CMV DNA titer rose remarkably and retinal lesions aggravated after IVG, ganciclovir was regarded to be ineffective and was replaced by foscarnet. The interval between repeated injections was one week. No patients received systemic anti-viral treatment.

All patients underwent a detailed ophthalmological examination at each visit, including BCVA (best corrected visual acuity), applanation tonometry, biomicroscopy of anterior segment and funduscopy. BCVA was measured using a decimal chart and was converted into LogMAR for computing purposes; a value of 2.6 LogMAR units was assigned for visual acuity of count fingers, 2.7 LogMAR units for hand movement, 2.8 LogMAR units for light perception and 2.9 LogMAR units for no light perception [11]. Fundoscopy was used to evaluate the extent of involvement at the time of diagnosis (0°–90°, 90°–180° and 180°–360°). The amount of CMV DNA load in aqueous humour, the total number of injections and the occurrence of complications were recorded in detail. Recurrence of CMVR was defined as the presence of new lesions after the initial retinitis had been resolved and was inactive. The occurrence of IVG-related complications was also recorded to evaluate the safety of the treatment.

Statistical analyses were carried out using SPSS software for Windows (version 22.0; IBM-SPSS, Chicago, IL, USA). The Mann-Whitney U test was used to compare the initial CMV loads between unilateral and bilateral CMVR. BCVA and CMV DNA loads at the baseline and the one-month visit were compared using the Wilcoxon signed-rank test. In patients with negative CMV titers after IVG, the relationship between the total number of IVG and the initial CMV DNA titers in aqueous humour was calculated using regression analysis, which was also used to calculate the relationship between baseline BCVA and final BCVA. The Kruskal–Wallis test was used to explore the relationship between the initial CMV titer and the retinal extent of CMV. *P*-values represented results for two-sided tests, with values less than 0.05 considered statistically significant.

## Results

The study cohort consisted of 24 patients (12 males and 12 females; 24 eyes total) with a mean age of 29.08 ± 14.16 years (range: 7–59 years). Among all included patients, 21 received hematopoietic stem cell transplantation therapy, while another 2 underwent chemotherapy due to the presence of a hematologic disease and 1 received thymectomy. Nine patients had bilateral disease. The mean CMV load in aqueous humour was (9.61 ± 17.67) × 10^4^ copies/mL in unilateral CMVR and (7.89 ± 10.51) × 10^4^ copies/mL in bilateral CMVR. No significant difference was found between the viral loads of the two groups (*P* = 0.817, Mann-Whitney U). The median follow-up time was 15 months (range: 3–42 months). The mean time-span from the beginning of the treatment to the retinitis quiescence was 3.74 ± 1.39 weeks (range: 1–7 weeks). No patient was lost in follow-up during the treatment except one patient died during the treatment. Table 1 lists the characteristics of the studied patients.

**Table 1:** Baseline Characteristics of Involved Patients

Characteristics	Patients (*n* = 24)
Age (year), mean ± SD (range)	29.08 ± 14.16 (7–59)
Gender, n (%)	
Male	12 (50)
Female	12 (50)
Eyes,n(%)	
Unilateral	15 (62.5)
Bilateral	9 (37.5)
Primary disease, n (%)	
Hematologic disease	23 (95.8)
Post HSCT	21 (87.5)
Chemotherapy	2 (8.3)
Thymectomy	1 (4.2)

*SD*, standard deviation; *HSCT*, hematopoietic stem cell transplantation therapy

**Fig. 1:**
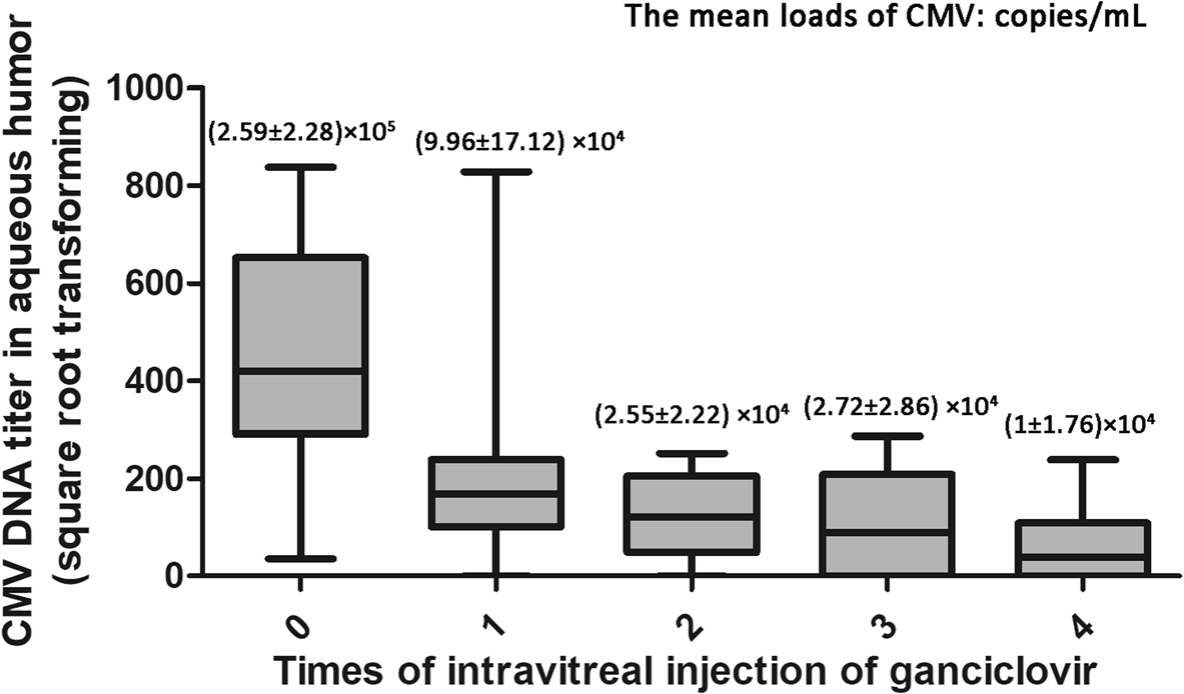
CMV DNA titer in aqueous humour of patients with CMVR during first four IVG once a week. CMV load decreased continuously during the procedure

The mean load of CMV in aqueous humour at baseline was (2.59 ± 2.28) × 10^5^ copies/mL, but it decreased significantly to (1 ± 1.76) × 10^4^ copies/mL after four IVG (*P* = 0.000, Wilcoxon signed-rank; Fig. 1). The mean number of IVGs was 3.46 ± 1.06 (range: 1–5 injections). The CMV DNA titer was tested negative in 6 eyes (25%) after 2 injections, in 11 eyes (45.8%) after 3 injections and in 17 eyes (70.8%) after 4 injections. The mean LogMAR BCVA was 0.87 ± 0.86 (range: 0–2.8) at baseline and 0.70 ± 0.79 (range: 0–2.9) at the one-month visit, which indicated no obvious improvement (*P* = 0.112, Wilcoxon signed-rank). The retinal extent of CMVR was ‘0°–90°’ in 9 eyes, ‘90°–180°’ in 6 eyes and ‘180°–360°’ in 9 eyes. The initial aqueous CMV DNA load was not statistically correlated with the CMVR extent (*P* = 0.098, Kruskal–Wallis).

**Fig. 2:**
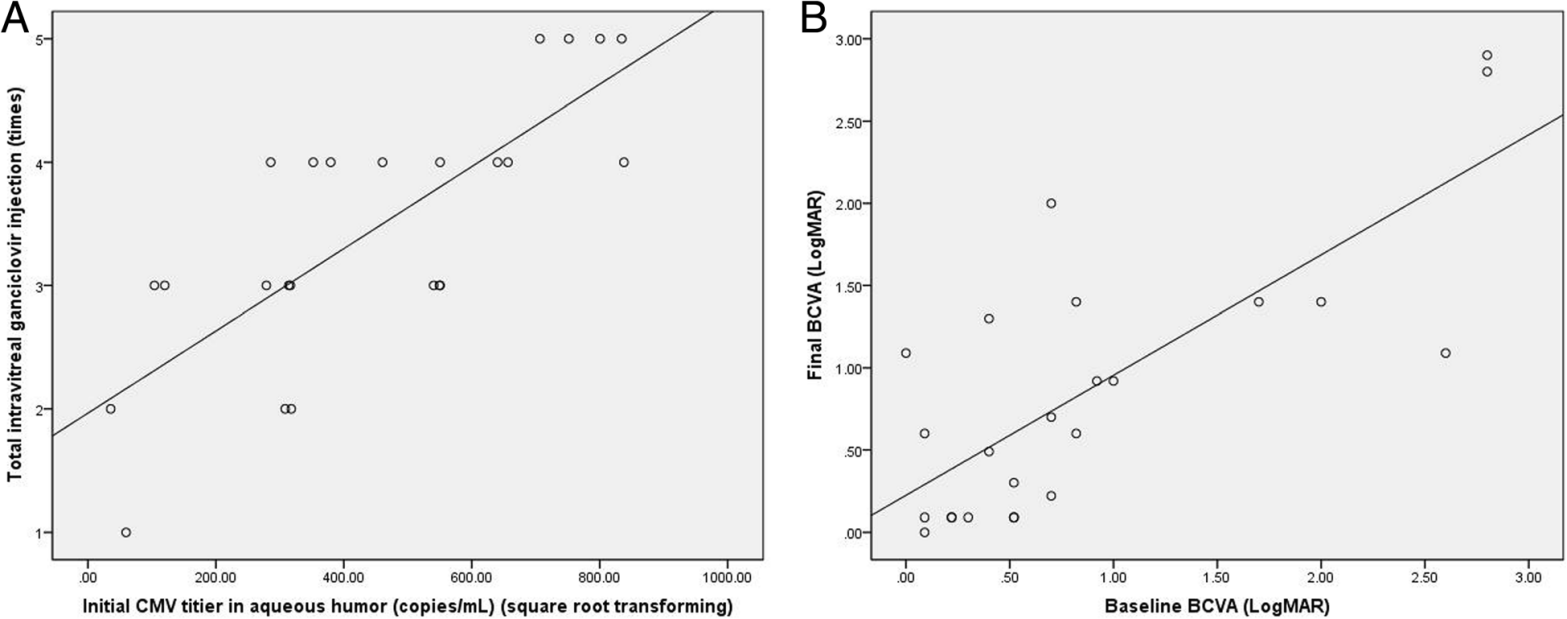
Regression analysis for total number of IVG and final BCVA. (**a**) Positive correlation between initial CMV DNA tiers in aqueous humour and total number of IVG (R^2^ = 0. 772, *P* = 0.000). (**b**) Positive correlation between baseline BCVA and final BCVA (R^2^ = 0.762,P = 0.000)

**Fig. 3:**
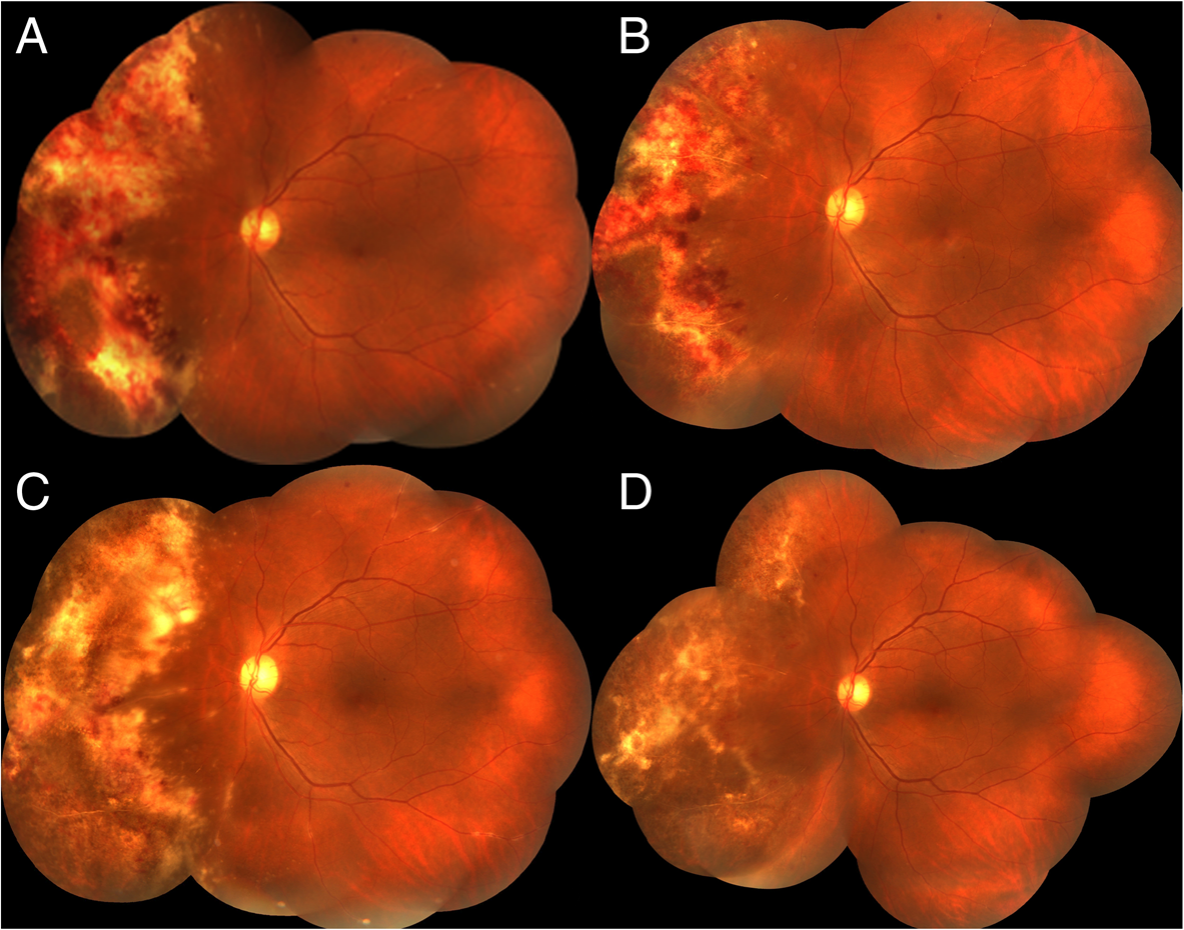
A case of ineffective intravitreal ganciclovir. **a** The active lesion and haemorrhage located in the nasal quadrant of retina. **b** After third IVG, the lesion size decreased with CMV DNA load in aqueous humour reduction. **c** After fourth IVG, the lesion size enlarged towards the posterior pole, and CMV DNA load in aqueous humour increased. **d** Intravitreal ganciclovir was replaced by foscarnet. After three injections, the lesion became inactive, and CMV DNA load in aqueous humour turned negative

Regression analysis revealed a positive correlation between initial CMV DNA titers in aqueous humour and the total number of IVG (Fig. 2a; R^2^ = 0.772, *P* = 0.000), as well as between baseline BCVA and final BCVA (Fig. 2b; R^2^ = 0.762,P = 0.000). Three eyes (12.5%) suffered recurrence of CMVR during the follow-up period. New lesions appeared in fundus examination, and CMV was redetected in aqueous humour several months (range: 2–6 months) after the regression of primary retinal lesions. In these patients, 3 mg/0.1 mL of IVG was restarted. CMVR relapsed in three eyes (12.5%), in which we detected a rise of CMV DNA load in aqueous humour and enlarged retinal lesions in fundus examination after several IVG. Intravitreal foscarnet of 2.4 mg was used as a substitute (Fig. 3). IVG was stopped in one eye because there was no light perception after three injections, while in another eye, IVG was stopped after five injections due to vitrectomy and silicone oil tamponade. One patient died due to cytomegaloviral pneumonitis one month after the CMV load turned negative. No retinal detachment due to intravitreal injection or endophthalmitis occurred during the treatment. No drug toxicity reaction was observed during the treatment.

## Discussion

Ganciclovir interferes with DNA polymerase, resulting in inhibition of viral replication. Previous studies have proven the efficacy of systematic ganciclovir in the treatment of CMVR in both HIV-positive and HIV-negative patients [12–15]. Vishnevskia-Dai V et al. proposed a repeated intravitreal use of ganciclovir and/or foscarnet in cases of macular or optic disc-threatening disease [16]. Side effects of systematic ganciclovir include renal failure, neutropenia or thrombocytopenia, which are commonly experienced in patients with myelosuppression [10, 17]. However, intravitreal use of ganciclovir has a low systemic exposure. Direct intraocular use also has the advantage of achieving therapeutic levels by circumventing the inefficiency of ganciclovir in crossing the blood-retina barrier [10]. In the previous study, we indicated that the major disadvantage of IVG was its little benefit for the fellow eye with no virus infection, but now we had impression that the progression of lesions in the fellow eye could be controlled well with close follow-up and timely treatment.

Compared to our previous study, the major difference in this treatment was the dose of intravitreal ganciclovir. We previously used 1 mg/time ganciclovir for each injection; in this study, the first injection dose was increased to 6 mg, followed by lower doses of 4.5 mg and 3 mg. Our present study suggests that this treatment regimen could also significantly reduced the CMV DNA titers in CMV-infected eyes. However, the percentage of patients with CMV turning negative after each injection did not increase in this study. On the contrary, in our present study, 25% of patients were CMV-negative after two injections, which was lower than what we reported in the previous study (47.8%) [10]. This difference could be attributed to the limited number of patients in both studies or the individual differences of sensitivity to ganciclovir. No significant improvement in BCVA was found in involved patients, even with the decrease of CMV in aqueous humour. The result was the same as in our previous study in which 1 mg IVG had been used as the treatment regimen. However, the final BCVA proved to be positively correlated with the baseline BCVA. We had the impression that eyes with good initial visual acuity (LogMAR BCVA less than 0.3) commonly had stable or improved BCVA at the final visit. According to our observations, most eyes with poor initial visual acuity had macular involvement (the macula is mainly responsible for photopic vision). We presume that the damage of photoreceptors by CMV infection is irreversible and irreparable, even if the lesion becomes inactive following the decrease of CMV DNA titers.

Joens et al. reported a mean number of 10 injections (range: 2–22) of ganciclovir with a dose of 2 mg/time, while in the study of Lu et al., the results were similar [18, 19]. In our study, the mean number of IVG was 3.46 ± 1.06, which was much less than in the above-mentioned studies. This discrepancy could be attributed to the higher dose of intravitreal ganciclovir in our study or the timely change to foscarnet when ganciclovir was ineffective in decreasing CMV DNA titer. We also found a strong relationship between the initial CMV DNA titers and the total number of IVG, which may be a predictive indicator of the treatment time spent in the following treatment.

In the present study, three eyes (12.5%) suffered recurrence of CMVR, which was lower than in the study of Lu et al [19] Several patients with recurred CMVR provided a history of ‘staying up late’ or ‘overworking’, from which we speculated that reduced immune function played a role in the reactivation of intraocular CMV. In the other three eyes, CMV DNA titers rose after several IVG, continuous lesion enlargement was observed and ganciclovir was replaced by intravitreal injections of foscarnet. A possible reason for the relapse was the non-response of the virus to ganciclovir, especially in our case because the initial dose of IVG was high. In these cases, the timely change to other antiviral agents is essential to prevent continuous damage to the retina by CMV.

Choopong et al. reported a case of sudden crystallization in vitreous cavity after 4 mg/0.04 mL ganciclovir, which caused optic atrophy [20]. It was speculated that high drug concentrations introduced in the eyeball globe lead to the sudden change of pH in the vitreous humour, which might cause crystallization of ganciclovir. The dose of ganciclovir we used was higher than as reported above, but the concentration was lower to prevent such an accident.

In sum, patients treated with weekly intravitreal gancivlovir injections (6 mg initially, then 4.5 mg, then 3 mg until cessation of therapy) experienced a significant reduction in anterior chamber CMV load. The mean total number of injections was less than that of previous studies. A positive correlation was proven between initial CMV DNA titers in aqueous humour and the total number of IVG, as well as between baseline BCVA and final BCVA.

Our study had certain inherent limitations due to its retrospective design. It also did not include a control group, so we cannot form any conclusions regarding the relative efficacy of this treatment over any other treatment. The sample size of this study was also relatively small, which may limit the statistical strength of the findings. Therefore, further studies with prospective, controlled design and a larger number of included patients are needed to confirm our conclusion.

## Abbreviations

BCVA: Best-corrected visual acuity
CMV: Cytomegalovirus
CMVR: CMV retinitis
IVG: Intravitreal injections of ganciclovir
PCR: Polymerase chain reaction
